# Perioperative Hypothermia in Medium- and Large-Volume Plastic Surgery: Clinical and Surgical Implications and Practical Prevention Strategies

**DOI:** 10.7759/cureus.112785

**Published:** 2026-07-16

**Authors:** Luiz Augusto Sousa Oliveira, Victor Da Costa Sacksida Valladão, Nélio Nunes Cabette Filho, Ligia Helena Mendes, Fábio André Zanella

**Affiliations:** 1 General Surgery, Fundação Hospitalar Santa Terezinha de Erechim, Erechim, BRA; 2 Plastic Surgery, Hospital Niterói D'Or, Serviço de Pós-Graduação em Cirurgia Plástica Prof. Ronaldo Pontes, Niterói, BRA; 3 Plastic Surgery, Hospital Nossa Senhora da Conceição, Grupo Hospitalar Conceição, Porto Alegre, BRA

**Keywords:** body contouring, forced-air warming, microsurgical flap, perioperative hypothermia, perioperative normothermia, plastic surgery, surgical quality indicator, surgical site infection

## Abstract

Inadvertent perioperative hypothermia (IPH), defined as a core temperature below 36 °C, is a common complication of elective procedures under general anesthesia and has traditionally been treated as an anesthetic concern. In medium- and large-volume plastic surgery, including abdominoplasty, large-volume liposuction, combined and postbariatric body contouring, and autologous and microsurgical reconstruction, wide body-surface exposure, cold tumescent solution, prolonged operative time, and combined anesthesia amplify this risk and make it directly relevant to surgical outcomes. This narrative review, structured around the Scale for the Assessment of Narrative Review Articles (SANRA) domains, synthesizes the pathophysiology; the wound-healing, flap, and microsurgical implications most relevant to plastic surgery; the subclinical hemodynamic consequences; the surgical outcomes; and practical prevention strategies for IPH, drawing on searches in PubMed/MEDLINE, Cochrane Library, SciELO, and Embase (1996-2024). Even mild IPH meaningfully increases the risks of surgical site infection, intraoperative bleeding, ischemic cardiac events, and delayed emergence; of particular concern in plastic surgery, it fosters a vasoconstricted, hypoperfused microcirculatory environment that threatens wound edges, random-pattern and pedicled flaps, and microsurgical anastomoses. Postoperative hypothermia is especially frequent after prolonged operations. Prewarming, forced-air warming, and fluid warming substantially reduce IPH incidence. We propose that perioperative normothermia be treated as an auditable institutional quality indicator, shared across the perioperative team, with the plastic surgeon contributing specific, protocol-driven actions.

## Introduction and background

Medium- and large-volume aesthetic and reconstructive plastic surgery encompasses abdominoplasty, lipoabdominoplasty, large-volume liposuction, mastopexy/mammoplasty, combined procedures, postbariatric body contouring, and microsurgical reconstruction. In these procedures, the convergence of wide body-surface exposure, cold tumescent infusion, prolonged operative time, and combined anesthesia, which abolishes central thermoregulatory defenses and promotes core-to-peripheral heat redistribution, creates conditions for substantial, progressive heat loss in the absence of an active normothermia protocol. Inadvertent perioperative hypothermia (IPH), defined as a core temperature below 36 °C at any pre-, intra-, or postoperative time-point [1], occurs in 20% to 70% of elective surgeries under general anesthesia [2,3]. This wide range reflects methodological heterogeneity across studies rather than true biological variation alone, as the reported incidence depends on the core-temperature threshold and measurement site used to define hypothermia, the timing of assessment, the type and duration of surgery, the anesthetic technique, and whether active warming was employed [2,3].

Traditionally, however, IPH has been approached primarily from the anesthetic side, even though many of its consequences fall within the surgical domain. This position is difficult to sustain given the current evidence. Even mild hypothermia (defined as a core temperature of 34-36 °C) is associated with major cardiovascular events [4], coagulopathy [5,6], increased transfusion requirements [7], surgical site infection [8], delayed emergence [9], and prolonged hospitalization [10]. In plastic surgery specifically, IPH is independently associated with postoperative complications [11,12]. Normothermia therefore has a legitimate place among auditable surgical-quality variables, alongside timely antibiotic prophylaxis, site marking, and patient safety measures [13,14].

Despite this evidence, adequately powered, plastic-surgery-specific trials with temperature endpoints remain scarce. This review therefore has two explicit objectives: first, to synthesize the clinical and surgical implications of IPH in medium- and large-volume plastic surgery, including its pathophysiology, subclinical hemodynamic consequences, wound and flap implications, and surgical outcomes, drawing on interventional evidence demonstrating that maintaining normothermia reduces these events; and second, to translate that evidence into a practical, stage-based prevention protocol for the whole perioperative team, in which the plastic surgeon contributes specific, auditable actions alongside the anesthesia and nursing teams.

## Review

Materials and methods

This is a narrative review following the methodological domains outlined in the Scale for the Assessment of Narrative Review Articles (SANRA) [15]. The narrative format was chosen to integrate mechanistic, monitoring, and preventive evidence into a clinically usable construct for the plastic surgeon, given that the heterogeneity of populations, anesthetic regimens, warming devices, and reported endpoints precludes meta-analytic synthesis. Although several systematic reviews and meta-analyses addressing individual aspects of perioperative hypothermia are available, each answers a single, narrowly framed question, such as active warming versus control for one specific outcome, whereas no existing systematic review spans the mechanistic, monitoring, and preventive continuum across the heterogeneous populations, anesthetic regimens, warming devices, and non-standardized endpoints relevant to plastic surgery. This multi-domain scope, rather than a single answerable question, is what a narrative synthesis is best suited to address [15].

Search Strategy

A structured search was performed in PubMed/MEDLINE, Cochrane Library, SciELO, and Embase between January 1996 and December 2024 (last update on 31 December 2024). The search combined MeSH terms and free-text descriptors: "perioperative hypothermia", "inadvertent perioperative hypothermia", "plastic surgery", "reconstructive surgery", "abdominoplasty", "body contouring", "microsurgical reconstruction", "thermoregulation", "forced-air warming", "prewarming", "tumescent solution", joined by AND/OR. Reference lists of major reviews and guidelines (NICE CG65, ASPAN, German S3) were hand-searched. This explicit, multi-database search reflects the literature-search quality domain of the SANRA instrument [15].

Eligibility

Included studies addressed the pathophysiology, monitoring, prevention, or treatment of IPH in adult or pediatric surgical patients; were randomized clinical trials, systematic reviews, meta-analyses, prospective cohorts, or guidelines from recognized societies; were published in English, Portuguese, or Spanish; and reported clinically meaningful endpoints, including core temperature, transfusion, cardiac events, surgical site infection, flap viability, length of stay, or post-anesthesia care unit (PACU) discharge. Preclinical studies, narrative editorials, isolated case reports, and studies without temperature endpoints were excluded, except when they were essential for pathophysiological grounding. Prioritizing the highest-quality evidence available for each thematic domain accords with the scientific rigor criteria of SANRA [15].

Evidence Appraisal and Synthesis

Two authors independently identified eligible studies by applying the predefined eligibility criteria, with preference given to systematic reviews, randomized controlled trials, and society guidelines. For each preventive intervention, a level of evidence was assigned according to the Oxford CEBM (1 = systematic review or RCT; 2 = cohort study; 3 = case-control study; 4 = case series; 5 = expert opinion). Because this is a narrative review, a formal risk-of-bias assessment of individual studies was not performed; instead, the strength of the underlying evidence is conveyed through these CEBM levels. The remaining findings, including pathophysiological, epidemiological, and outcome-related data, were integrated narratively across thematic domains: thermoregulatory physiology, plastic-surgery-specific risk factors, wound/flap implications, subclinical consequences, monitoring/prevention, and treatment. A practical decision algorithm was developed to translate the synthesized evidence into operative-room practice. This graded, thematically integrated synthesis follows the scientific-reasoning and data-presentation domains of SANRA [15].

Pathophysiology of intraoperative thermoregulation

The body maintains core temperature within a narrow range of 36.5-37.5 °C through a hypothalamic regulatory system whose interthreshold range, defined as the range between vasodilation/sweating and vasoconstriction/shivering, is approximately 0.2 °C while awake [1,16]. General anesthesia widens this range to about 4 °C, abolishing autonomic defenses against cold during the first hour after induction [16].

**Figure 1:**
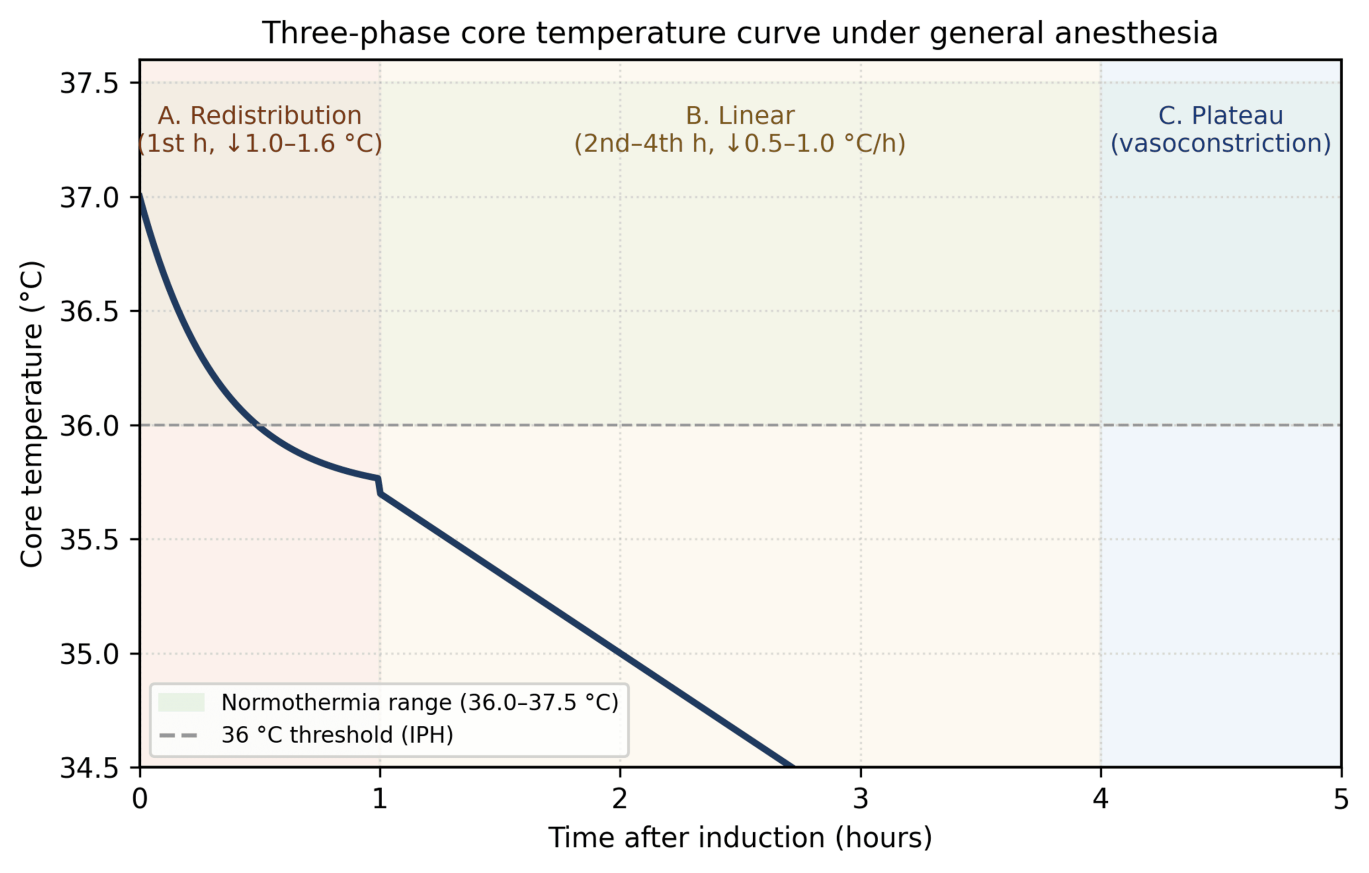
Three-phase curve of core temperature under general anesthesia (A) Redistribution phase during the first hour; (B) linear phase of loss greater than production between hours 2 and 4; (C) plateau phase due to thermoregulatory vasoconstriction Adapted from Sessler [16]

Core temperature decline under general anesthesia follows a characteristic three-phase pattern (Figure 1). In the redistribution phase, during the first hour, core temperature falls by 1.0-1.6 °C as anesthetic vasodilation drives central-to-peripheral heat redistribution. A subsequent linear phase, between the second and fourth hours, produces a slower decline of 0.5-1.0 °C per hour, as environmental heat loss exceeds metabolic production. A final plateau phase stabilizes the temperature at 33-35 °C, sustained by thermoregulatory vasoconstriction that is only partially preserved under anesthesia [1,16].

Neuraxial blockade aggravates this scenario by preventing vasoconstriction in the sublesional territory and abolishing afferent thermal perception, causing a further decrease in temperature without triggering shivering [16]. The combination of general anesthesia with epidural or spinal anesthesia, common in abdominoplasty and combined procedures, substantially increases the risk of IPH [16]. Tumescent solution infused at room temperature (≈ 22 °C) at volumes greater than 2-3 L acts as a true cooling fluid and can induce clinically significant hypothermia even in short procedures [12,17].

Plastic surgery-specific risk factors

Risk factors can be stratified into three domains: surgical (primary in this context), patient, and anesthetic (Table 1). In a prospective registry of 308 postbariatric body-contouring patients (mean operative time of 4.7 h), the mean minimum intraoperative core temperature was 35.6 °C, with more than half of the patients reaching temperatures below 36 °C at some point under the institution's routine perioperative care [12]. That study did not itemize the specific warming devices used during the observation period; its findings led the authors to subsequently adopt a dedicated protocol including preoperative warming, an elevated operating-room temperature, and routine warmed fluids [12]. In a Brazilian case series of major surgery, postoperative hypothermia was documented in ~39% of patients, with a significant association with procedures exceeding four hours (p = 0.018) [18]. This temporal threshold coincides with the cut-off already identified as an independent predictor of complications in Brazilian plastic surgery [19]. Although not specific to plastic surgery, this Brazilian case series reinforces the relevance of operative time as a marker of thermal risk in our context.

**Table 1:** Risk stratification for iIPH in plastic surgery IPH: inadvertent perioperative hypothermia; BMI: body mass index; ASA: American Society of Anesthesiologists

Domain	Risk factors
Surgical	Operative time > 2 h (particularly > 4 h); wide body-surface exposure (abdominoplasty, lipoabdominoplasty, body contouring, large-volume liposuction, mastopexy/mammoplasty, combined procedures); tumescent volume > 2 L; extensive irrigation; microsurgical reconstruction
Patient	BMI < 20 or > 35 kg/m²; age > 60 years; ASA physical status ≥ III; female sex; low muscle mass; cachexia; prior burns
Anesthetic	General anesthesia with volatile agents; combined general + neuraxial; prolonged sedation; infusion of unwarmed fluids

Clinical and surgical implications of perioperative hypothermia

Five vectors connect IPH to outcomes of direct surgical relevance in medium- and large-volume plastic surgery, and each has been shown in randomized or controlled studies to improve when normothermia is maintained. First, mild hypothermia increases intraoperative bleeding by roughly 16% [5,6], thereby prolonging operative time, worsening the surgical field, and increasing transfusion requirements [7]. Second, it roughly triples the rate of surgical site infection [8], with downstream consequences including reoperation for drainage, dehiscence, loss of the aesthetic result, and potential litigation. Third, it increases the risk of major cardiac events (RR 2.2, 95% CI 1.1-4.7) [4], which may force cancellation of subsequent stages and prolong hospital stay, thereby increasing morbidity and mortality. Fourth, it delays emergence by 40-90 minutes [9], thereby impairing operating-room turnover and reducing surgical-suite productivity. Fifth, it compromises wounds and flaps; given the centrality of this outcome in plastic surgery, it is addressed in a dedicated subsection below. From this perspective, normothermia ceases to be an ancillary concern and joins the surgical-safety bundle for medium- and large-volume plastic surgery, alongside site marking, antibiotic prophylaxis, and thromboprophylaxis [13,14].

Implications for wound healing, flaps, and microsurgery

Wound integrity, flap viability, and microsurgical perfusion are the outcomes most distinctively at stake in plastic surgery, and they are also among the most temperature-sensitive. Because reconstructive plastic surgery routinely depends on marginally perfused tissue - long random-pattern flaps, pedicled musculocutaneous flaps, and free-tissue transfer - the vasoconstrictive and rheologic effects of even mild hypothermia translate more directly into flap compromise here than in most other surgical settings [11,17].

Surgical Wound

Thermoregulatory vasoconstriction reduces tissue oxygen tension and impairs neutrophil oxidative function, creating an environment unfavorable to healing and amplifying the risk of surgical site infection - the seminal study by Kurz et al. [8] demonstrated a tripling of infection rates in hypothermic compared with normothermic patients (19% vs. 6%; p = 0.009), and wound tissue oxygen tension is recognized as an independent predictor of wound infection in general surgical populations [20]. In long incisions, wide undermining, and suture lines under tension - abdominoplasty, lipoabdominoplasty, mastopexy/mammoplasty, combined procedures, and postbariatric patients - impaired healing and increased risk of marginal dehiscence compound the picture. In plastic surgery, specific evidence remains largely indirect, but the mechanistic plausibility is robust [11,17].

Local and Random-Pattern Flaps

Vasoconstriction reduces cutaneous flow, with greater vulnerability in distal regions and random-pattern designs. The effect is potentiated when tension, undermining, smoking, diabetes, or prior radiotherapy coexist. The most frequent practical consequences are edge suffering, epidermolysis, partial necrosis, prolonged dressings, and the need for touch-ups or reoperations [16,17].

Pedicled Flaps 

Vasoconstriction reduces pedicle flow and, combined with increased blood viscosity and reduced erythrocyte deformability, impairs local microcirculation. Postoperative shivering raises metabolic and oxygen demand at the most critical moment for flap survival. In large autologous breast reconstruction (pedicled TRAM, latissimus dorsi) and in major trunk or lower-limb reconstruction with fasciocutaneous or musculocutaneous flaps - where a single flap may carry the entire reconstruction - maintenance of normothermia is an adjunctive but non-trivial measure for tissue protection and for reliable clinical flap monitoring [16,17].

Free Flaps and Microsurgery

In free-tissue transfer, including DIEP and free-TRAM breast reconstruction, head-and-neck reconstruction, and limb salvage, hypothermia-induced vasoconstriction and microvascular spasm reduce capillary flow and foster a microcirculatory environment unfavorable to a freshly sutured anastomosis, thereby compounding the thrombotic vulnerability of the first postoperative hours. Because a single episode of flap loss can result in total reconstructive failure, reoperation, and a markedly worse outcome for the patient, normothermia is incorporated into ERAS protocols for microsurgical reconstruction [21,22] and is regarded as a relevant measure for preserving tissue perfusion and reducing perioperative morbidity [23]. A summary by surgical scenario, including mechanism, practical consequence, and preventive strategy, is presented below (Table 2).

**Table 2:** Implications of hypothermia by surgical scenario in plastic surgery: mechanism, practical consequences, and preventive conduct ERAS: Enhanced Recovery After Surgery; OR: operating room; core T: core temperature

Surgical scenario	Effect of hypothermia	Practical consequence	Preventive conduct
Abdominoplasty/lipoabdominoplasty/postbariatric	Vasoconstriction + coagulopathy + cold high-volume tumescent	Hematoma, dehiscence, marginal necrosis	Prewarming, warmed tumescent, minimal exposure, continuous monitoring
Local/random-pattern flap	Reduced distal perfusion	Edge suffering, epidermolysis, partial necrosis	Continuous normothermia, avoid cold OR, monitor core T
Pedicled flap	Reduced pedicle flow; ↑ blood viscosity	Congestion/suffering	Active intra- and postoperative warming, clinical vigilance
Free flap/microsurgery	Vasoconstriction; microcirculation unfavorable to anastomosis	Anastomosis revision, partial or total loss	Normothermia as ERAS target, intra- and postoperatively
Skin graft	Reduced bed perfusion	Impaired integration, infection	Warming, humidity control, warmed irrigation

Subclinical hemodynamic consequences

Beyond the overt complications described above, IPH triggers a set of hemodynamic alterations that frequently go unnoticed under standard intraoperative monitoring. Although subclinical, these changes increase cardiac strain and contribute to postoperative morbidity, and the most relevant are detailed below [16,24].

Peripheral Vasoconstriction and Increased Afterload

Thermoregulatory vasoconstriction raises systemic vascular resistance by up to 30-40%, increasing afterload and left ventricular workload [16,24]. In patients with limited myocardial reserve, this increment may precipitate silent ischemia.

Increased Myocardial Oxygen Consumption 

Postanesthetic shivering raises O₂ consumption by up to 200-400% above baseline [24]. In an extubated, still hypothermic patient, this peak coincides with the period of greatest hemodynamic lability and cardiac risk [4].

Electrophysiologic Alterations

Even mild hypothermia is associated with QT prolongation, sinus bradycardia, and - in moderate hypothermia - the classical Osborn J wave [25]. In predisposed patients, these may progress to ventricular arrhythmias.

Hemostatic Dysfunction

Hypothermia simultaneously compromises the three hemostatic pillars [5,6,26]: platelet function (reduced thromboxane-A₂-dependent adhesion/aggregation), coagulation cascade (at 33 °C, enzymatic activity ≈ 50% of normothermic values, though laboratory coagulograms run at 37 °C may appear normal - an important interpretive risk), and fibrinolysis (relative hyperfibrinolysis). Rajagopalan et al. [5] showed that mild hypothermia (≈ 1 °C below normal) increases blood loss by ~16% and the relative transfusion risk by 22%. This coagulopathy paradoxically coexists with increased late thrombotic risk, requiring careful balance with thromboprophylaxis in major plastic surgery [27].

Altered Pharmacokinetics

Hepatic drug metabolism is temperature-dependent, so clearance of the usual anesthetic agents falls by roughly 5-10% per °C below normal [16]; the practical surgical consequence is delayed emergence [9] and prolonged PACU occupancy that blocks operating-room turnover.

Frequently confused conceptual distinctions

Five distinctions structure the clinical reasoning and help avoid recurrent errors. First, IPH is not therapeutic hypothermia: IPH is a preventable adverse event, whereas therapeutic hypothermia (for cardiac arrest or neuroprotection) is a controlled intervention with specific targets and populations, and it does not justify tolerating IPH. Second, hypothermia is graded as mild (34-36 °C), moderate (32-34 °C), or severe (< 32 °C); even the mild form, the focus of this review, is sufficient to mediate all the surgical outcomes discussed, so waiting for shivering or bradycardia before intervening is already too late. Third, thermoregulatory vasoconstriction must be distinguished from pharmacologic vasoconstriction: the former is involuntary, prolonged, and compromises tissue and flap perfusion [21], whereas the latter is induced (e.g., by tumescent adrenaline) and has a distinct profile. Fourth, target normothermia (≥ 36 °C) is not iatrogenic hyperthermia (> 38 °C): active warming without continuous monitoring may overheat the patient, particularly in pediatric or burn cases, which is why central sensing is mandatory [16]. Fifth, core temperature must be distinguished from peripheral temperature: axillary and exposed-skin readings do not reflect core temperature and should not guide clinical decisions; the intraoperative gold standard is distal esophageal temperature measurement, with validated alternatives including nasopharyngeal, bladder (when urine output > 0.5 mL/kg/h), and forehead zero-heat-flux monitoring [16,28].

Surgical outcomes: magnitude, severity, and time window

Surgical outcomes associated with IPH can be consolidated along a single axis: effect magnitude, Clavien-Dindo severity, time window, and implication for the surgeon (Table 3). In microsurgery, hypothermia-induced vasoconstriction compromises free-flap perfusion, and normothermia is a central component of ERAS protocols for microsurgical reconstruction [21,22].

**Table 3:** Surgical outcomes associated with IPH: magnitude, severity (Clavien-Dindo), time window, and implications for the surgeon IPH: inadvertent perioperative hypothermia; RR: relative risk; CI: confidence interval; OR: odds ratio; PACU: post-anesthesia care unit; ICU: intensive care unit; CEBM: Oxford Centre for Evidence-Based Medicine levels of evidence (1 = systematic review/RCT; 2 = cohort; 3 = case-control; 4 = case series; 5 = expert opinion); RCT: randomized controlled trial

Surgical outcome	Effect magnitude	Severity (Clavien-Dindo)	Window	Implication for the surgeon	Level of evidence (CEBM)
Ischemic cardiac events	RR 2.2 (95% CI 1.1–4.7) [4]	IV	0–72 h	Cancellation of subsequent stages; ICU; mortality	1 (RCT)
Intraoperative bleeding	+16% (mean) [5]	II	Intraoperative	Prolonged operative time; unfavorable field	1 (meta-analysis of RCTs)
Transfusion requirement	RR ≈ 1.22 [5]	II	0–24 h	Transfusion logistics; additional risk	1 (meta-analysis of RCTs)
Surgical site infection	RR ≈ 3.0 [8]	II–III	3–30 days	Reoperation; dehiscence; aesthetic loss	1 (RCT)
Delayed emergence/PACU stay	+40 to +90 min [9]	I–II	0–6 h	Blocked OR turnover	1 (RCT)
Microsurgical flap compromise	OR ↑ in reconstruction series [21,23]	III–IV	0–14 days	Anastomosis revision; flap loss	2–4 (observational)
Prolonged hospitalization/readmission	Adjusted OR 1.3–1.8 [11,12]	II	≤ 30 days	Cumulative effect of complications above	2 (cohort)

Temperature monitoring

NICE CG65 recommends continuous core-temperature monitoring in any surgery lasting > 30 minutes under general anesthesia [28]. The distal esophageal site is the intraoperative gold standard in intubated patients; the nasopharyngeal route is an adequate alternative, and the bladder is reliable when urine output exceeds 0.5 mL/kg/h, whereas contact tympanic readings are artifact-prone and forehead zero-heat-flux provides a noninvasive option validated both intra- and postoperatively [16,28]. Axillary and exposed-skin measurements are inadequate for clinical decision-making and should not replace core temperature [16,28].

Three-stage prevention: roles within the perioperative team

**Figure 2:**
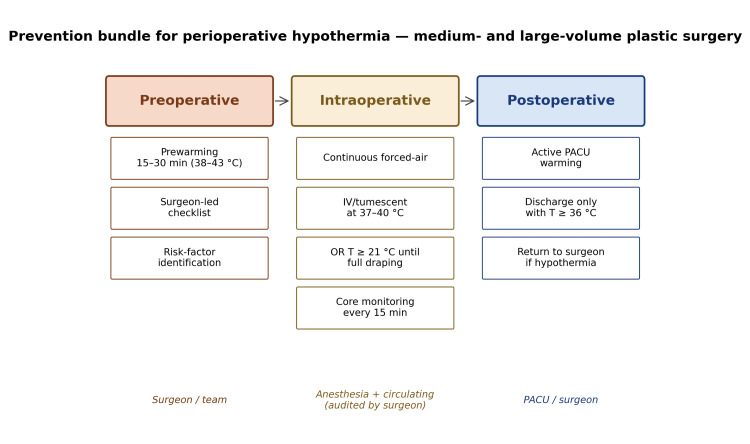
Prevention bundle for IPH in medium- and large-volume plastic surgery Preoperative, intraoperative, and postoperative axes, with explicit allocation of roles among the perioperative team IPH: inadvertent perioperative hypothermia; PACU: post-anesthesia care unit

Prevention is more effective and more cost-effective than treatment. Monitoring and execution of thermal measures remain under anesthetic and operating-room team coordination; the plastic surgeon in charge of the case is responsible for including normothermia in the procedural safety checklist and ensuring that the variable is tracked as an institutional quality outcome. A three-stage approach is recommended (Figure 2) [28-30].

Preoperative

Prewarming for 15 to 30 minutes with a forced-air blanket at 38-43 °C before induction is the single intervention with the greatest impact, acting on thermal redistribution [31,32]. A 2024 meta-analysis confirms significant reduction of IPH incidence with structured prewarming [33]. The earlier Cochrane meta-analysis of active body-surface warming reported a significant reduction in surgical site infection (RR 0.36, 95% CI 0.20-0.66), albeit based on low-quality evidence from 3 RCTs (589 participants) [34].

Intraoperative

Intraoperatively, continuous forced-air warming is the modality with the broadest evidence base and the widest availability [34,35]. Intravenous fluids and tumescent solution should be warmed to 37-40 °C, especially when the anticipated volume exceeds 1 L [12,17,36]. The operating-room temperature should be kept at ≥ 21 °C until full draping [28], with body-surface exposure limited to the minimum required and reflective thermal drapes used when applicable. In very prolonged surgeries, humidification and warming of anesthetic gases provide additional benefit [30]. Resistive electric blankets or circulating-water mattresses are useful alternatives or complements to forced air [37].

Postoperative

Postoperatively, temperature monitoring should continue until core temperature reaches ≥ 36 °C, with active warming applied as needed; maintaining normothermia at this stage limits shivering-related oxygen demand and supports hemostatic and wound recovery [28,29]. The level of evidence supporting each preventive intervention across the three stages is summarized in Table 4. 

**Table 4:** Evidence summary by preventive intervention (Oxford CEBM levels) CEBM: Centre for Evidence-Based Medicine; RCT: randomized controlled trial; IPH: inadvertent perioperative hypothermia; core T: core temperature; PACU: post-anesthesia care unit; NICE: National Institute for Health and Care Excellence; ASPAN: American Society of PeriAnesthesia Nurses

Intervention	CEBM level	Magnitude/key effect	Refs.
Active prewarming (15–30 min)	1 (RCTs + meta-analyses)	↓ IPH incidence; 2024 meta-analysis confirms significant reduction; Cochrane: RR 0.36 (0.20–0.66)	[31,32,33,34]
Intraoperative forced-air warming	1 (multiple RCTs + meta-analyses)	Maintains core T 0.5–1.0 °C above control	[3,29,35,37]
Warming of IV/tumescent fluids	1 (Cochrane)	↓ core T drop by ~0.5 °C when > 1 L	[12,17,36]
Operating-room T ≥ 21 °C until draping	1 (NICE/German S3)	Reduces redistribution loss	[26,28]
Reflective drapes/minimal exposure	3–4 (series; physiologic rationale)	Adjunctive; no RCT specific to plastic surgery	[17,38]
Humidification/warming of anesthetic gases	2 (cohort + guideline)	Marginal; useful > 4 h	[30]
Resistive blankets/water-circulating mattresses	1 (RCT)	Equivalent to forced air	[37]
Active postoperative warming until T ≥ 36 °C	1 (ASPAN/NICE)	Reduces PACU time and shivering	[28,30]

Treatment of established hypothermia

When core temperature remains < 36 °C despite preventive measures, recommended management proceeds along several lines. Active surface warming is intensified (forced air at 43 °C), and all infused fluids are warmed; in selected cases, extubation may be deferred until adequate thermal recovery. Shivering is managed pharmacologically, with meperidine 12.5-25 mg IV the most effective option and dexmedetomidine or clonidine as alternatives [38]. Clinical coagulation and the ECG warrant heightened attention, and at temperatures < 34 °C intravascular or high-flow warming devices should be considered [28,30].

Practical algorithm and auditable checklist

**Figure 3:**
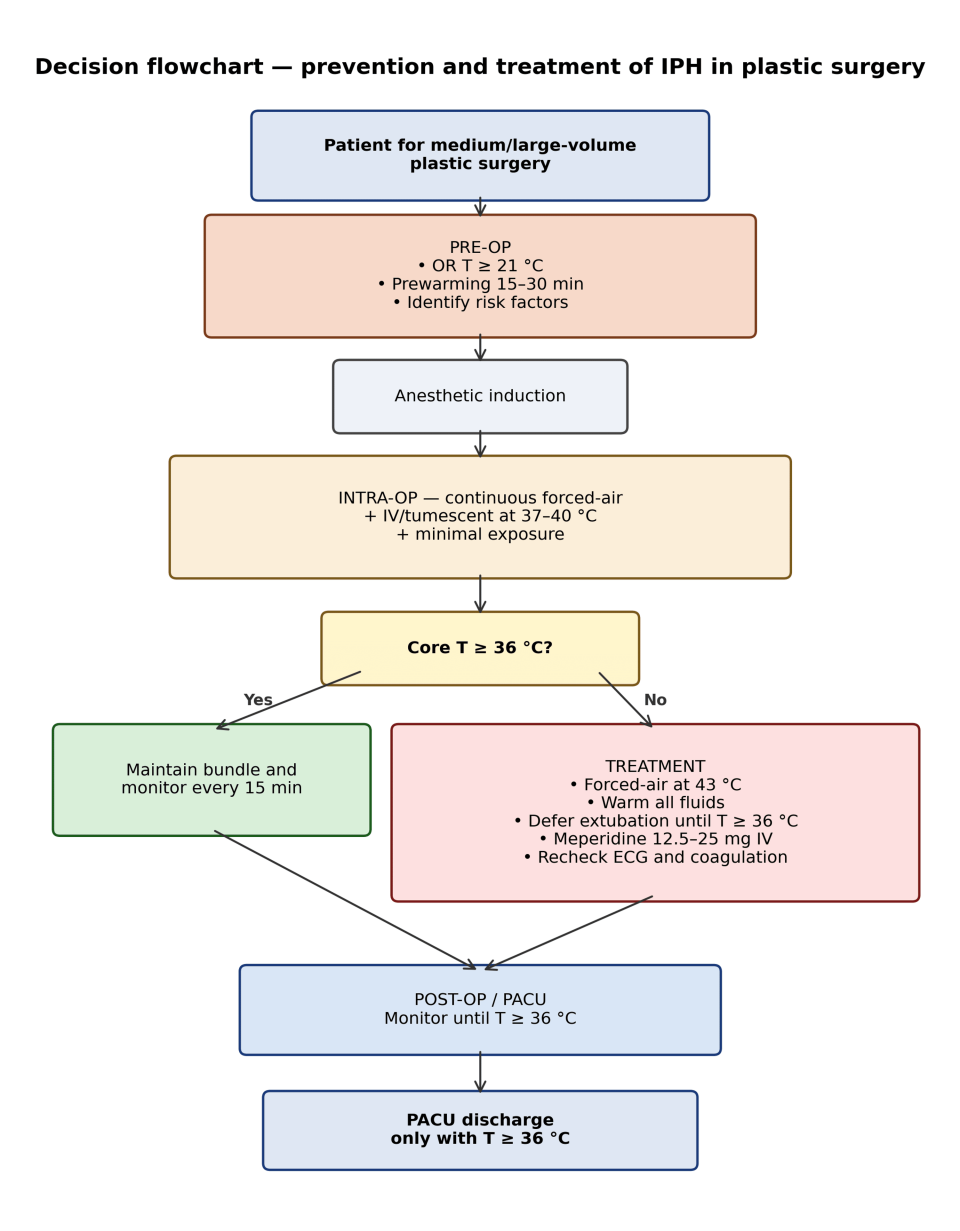
Decision-making flowchart for the prevention and treatment of IPH in medium- and large-volume plastic surgery Stepwise algorithm across the preoperative, intraoperative, and postoperative stages, with core T ≥ 36 °C as the recommended PACU discharge criterion IPH: inadvertent perioperative hypothermia; core T: core temperature; PACU: post-anesthesia care unit

The decision algorithm (Figure 3) summarizes, across the three perioperative stages, the actions recommended by the model proposed in this review. The corresponding auditable checklist (Table 5) is intended for direct use by the operating-room team and for validation by the responsible surgeon, with core T ≥ 36 °C recommended as the safety criterion for PACU discharge, in accordance with institutional protocol [28,29].

**Table 5:** Auditable normothermia checklist for medium- and large-volume plastic surgery: items and responsible team member OR: operating room; core T: core temperature; PACU: post-anesthesia care unit

Auditable item	Responsible
Normothermia verified in the surgical safety checklist/pre-incision time-out	Surgeon
Warmed tumescent/irrigation solution (37–40 °C) specified in the operative plan	Surgeon/anesthesia
Ambient temperature ≥ 21 °C verified	Circulating nurse/OR team
Prewarming ≥ 15 min before induction	Anesthesia/circulating nurse
Core temperature sensor in place	Anesthesia
Forced-air blanket operating	Anesthesia/circulating nurse
Body-surface exposure minimized and operative time optimized (staging of very long combined cases)	Surgeon
IV fluids and tumescent solution warmed to 37–40 °C	Anesthesia/circulating nurse
Temperature reassessed every 15 min and charted	Anesthesia
Core T ≥ 36 °C recommended as PACU discharge criterion (per institutional protocol)	Surgeon/PACU

Special populations

Pediatric and burn surgery warrant complementary mention. In pediatric plastic surgery, including burn debridement and grafting, craniofacial reconstruction, and cleft lip and palate repair, the high surface-to-mass ratio and thermoregulatory immaturity require active prewarming, pediatric forced-air warming, warmed irrigation, and an operating-room temperature of ≥ 23 °C; core temperature monitoring is mandatory in any procedure > 30 minutes [39]. In acute burn surgery, continuous evaporative losses from the wound bed produce IPH in up to 70% of cases despite aggressive warming, supporting staged excisions and warmed colloid resuscitation [17]. Postbariatric body contouring and microsurgery, given their centrality in aesthetic and reconstructive plastic surgery, are addressed in the subsection on wound healing, flaps, and microsurgery.

Limitations of this review

Several limitations should be considered when interpreting the findings of this review. First, this is a narrative review and does not replace the quantitative synthesis of a systematic review with meta-analysis; the heterogeneity of populations, anesthetic regimens, warming devices, and reported endpoints in the primary literature, particularly within plastic surgery, precluded a robust specialty-specific meta-analytic aggregation at the date of this review. Second, narrative selection carries an inherent risk of bias, which is only partially mitigated by the SANRA-domain structuring and by the explicit Oxford CEBM grading applied to each preventive intervention. Third, much of the flap-specific evidence rests on mechanistic plausibility and on extrapolation from general-surgery and trauma cohorts, because adequately powered plastic-surgery trials with temperature endpoints are still lacking, representing a gap this review makes explicit rather than conceals. Fourth, the cited guidelines (NICE CG65, ASPAN, German S3) reflect North Atlantic practice and may require adaptation to Brazilian technological and population profiles, with this adaptation being only partially anchored by the available national data [18,19]. These constraints define the boundary within which the proposed normothermia model should be interpreted and prospectively validated.

## Conclusions

IPH is prevalent, preventable, and cost-effective to combat. In medium- and large-volume plastic surgery, the convergence of surgical factors, including exposure, tumescent solution, operative time, and combined anesthesia, demands an active, protocol-driven, and auditable posture. Its consequences, often silent in the operating room, translate into bleeding, infection, cardiac events, wound and flap compromise, and prolonged hospitalization, all of which are outcomes of direct surgical relevance. There is also a relevant pathophysiological interface between IPH and perioperative hemostatic disorders, in which preventing hypothermia adds to, rather than opposes, appropriate thromboprophylaxis: normothermia preserves intraoperative hemostatic function and reduces venous stasis from vasoconstriction, contributing to a more favorable thrombotic profile in the early postoperative period. We therefore propose the consolidation of perioperative normothermia as an auditable institutional quality indicator, alongside timely antibiotic prophylaxis, site marking, and thromboprophylaxis, as a shared perioperative responsibility to which the plastic surgeon contributes specific, protocol-driven actions. Temperature management is already well established within anesthesia practice; this review makes its surgical stakes explicit and defines the complementary actions that the surgical team can audit and own.

## References

[1] Sessler DI (2016). Perioperative thermoregulation and heat balance. Lancet.

[2] Yi J, Lei Y, Xu S (2017). Intraoperative hypothermia and its clinical outcomes in patients undergoing general anesthesia: national study in China. PLoS One.

[3] Sun Z, Honar H, Sessler DI (2015). Intraoperative core temperature patterns, transfusion requirement, and hospital duration in patients warmed with forced air. Anesthesiology.

[4] Frank SM, Fleisher LA, Breslow MJ, Higgins MS, Olson KF, Kelly S, Beattie C (1997). Perioperative maintenance of normothermia reduces the incidence of morbid cardiac events. A randomized clinical trial. JAMA.

[5] Rajagopalan S, Mascha E, Na J, Sessler DI (2008). The effects of mild perioperative hypothermia on blood loss and transfusion requirement. Anesthesiology.

[6] Schmied H, Kurz A, Sessler DI, Kozek S, Reiter A (1996). Mild hypothermia increases blood loss and transfusion requirements during total hip arthroplasty. Lancet.

[7] Wolberg AS, Meng ZH, Monroe DM 3rd, Hoffman M (2004). A systematic evaluation of the effect of temperature on coagulation enzyme activity and platelet function. J Trauma.

[8] Kurz A, Sessler DI, Lenhardt R (1996). Perioperative normothermia to reduce the incidence of surgical-wound infection and shorten hospitalization. Study of Wound Infection and Temperature Group. N Engl J Med.

[9] Lenhardt R, Marker E, Goll V (1997). Mild intraoperative hypothermia prolongs postanesthetic recovery. Anesthesiology.

[10] Billeter AT, Hohmann SF, Druen D, Cannon R, Polk HC Jr (2014). Unintentional perioperative hypothermia is associated with severe complications and high mortality in elective operations. Surgery.

[11] Constantine RS, Kenkel M, Hein RE, Cortez R, Anigian K, Davis KE, Kenkel JM (2015). The impact of perioperative hypothermia on plastic surgery outcomes: a multivariate logistic regression of 1062 cases. Aesthet Surg J.

[12] Coon D, Michaels J 5th, Gusenoff JA, Chong T, Purnell C, Rubin JP (2012). Hypothermia and complications in postbariatric body contouring. Plast Reconstr Surg.

[13] Speth J (2025). Guidelines in practice: patient temperature management. AORN J.

[14] Saucedo OH, Ribeiro ER, Muller JC, Coelho IC (2020). Patient safety in plastic surgery: a systematic review. Rev Bras Cir Plást.

[15] Baethge C, Goldbeck-Wood S, Mertens S (2019). SANRA-a scale for the quality assessment of narrative review articles. Res Integr Peer Rev.

[16] Sessler DI (2000). Perioperative heat balance. Anesthesiology.

[17] Young VL, Watson ME (2006). Prevention of perioperative hypothermia in plastic surgery. Aesthet Surg J.

[18] Barbosa ML, Ximenes Júnior L, Rossi FA, Silva CR (2025). Postoperative hypothermia in the post-anesthesia care unit (Article in Portuguese). Braz J Health Rev.

[19] Saldanha OR, Salles AG, Llaverías F, Saldanha Filho OR, Saldanha CB (2014). Predictive factors for complications in plastic surgery procedures: suggested safety scores. Rev Bras Cir Plást.

[20] Hopf HW, Hunt TK, West JM (1997). Wound tissue oxygen tension predicts the risk of wound infection in surgical patients. Arch Surg.

[21] Astanehe A, Temple-Oberle C, Nielsen M (2018). An enhanced recovery after surgery pathway for microvascular breast reconstruction is safe and effective. Plast Reconstr Surg Glob Open.

[22] Temple-Oberle C, Shea-Budgell MA, Tan M (2017). Consensus review of optimal perioperative care in breast reconstruction: Enhanced Recovery after Surgery (ERAS) Society recommendations. Plast Reconstr Surg.

[23] Sumer BD, Myers LL, Leach J, Truelson JM (2009). Correlation between intraoperative hypothermia and perioperative morbidity in patients with head and neck cancer. Arch Otolaryngol Head Neck Surg.

[24] Frank SM, Fleisher LA, Olson KF (1995). Multivariate determinants of early postoperative oxygen consumption in elderly patients. Effects of shivering, body temperature, and gender. Anesthesiology.

[25] Mallet ML (2002). Pathophysiology of accidental hypothermia. QJM.

[26] Torossian A, Bräuer A, Höcker J, Bein B, Wulf H, Horn EP (2015). Preventing inadvertent perioperative hypothermia. Dtsch Arztebl Int.

[27] Sousa Oliveira LA, Helena Mendes L, Cabette Filho NN, da Costa Sacksida Valladão V (2026). Thromboprophylaxis in intermediate and major plastic surgery procedures: a narrative review and proposal of the TROMBO-PLAST integrated decision-making model. Cureus.

[28] (2026). National Institute for Health and Care Excellence: hypothermia: prevention and management in adults having surgery. Clinical guideline CG65. https://www.nice.org.uk/guidance/cg65.

[29] Hooper VD, Chard R, Clifford T (2010). ASPAN's evidence-based clinical practice guideline for the promotion of perioperative normothermia: second edition. J Perianesth Nurs.

[30] Forbes SS, Eskicioglu C, Nathens AB, Fenech DS, Laflamme C, McLean RF, McLeod RS (2009). Evidence-based guidelines for prevention of perioperative hypothermia. J Am Coll Surg.

[31] Andrzejowski J, Hoyle J, Eapen G, Turnbull D (2008). Effect of prewarming on post-induction core temperature and the incidence of inadvertent perioperative hypothermia in patients undergoing general anaesthesia. Br J Anaesth.

[32] Horn EP, Bein B, Böhm R, Steinfath M, Sahili N, Höcker J (2012). The effect of short time periods of pre-operative warming in the prevention of peri-operative hypothermia. Anaesthesia.

[33] Uçak A, Tat Çatal A, Karadağ E, Cebeci F (2024). The effect of prewarming on perioperative hypothermia: a systematic review and meta-analysis of randomized controlled studies. J Perianesth Nurs.

[34] Madrid E, Urrútia G, Roqué i Figuls M (2016). Active body surface warming systems for preventing complications caused by inadvertent perioperative hypothermia in adults. Cochrane Database Syst Rev.

[35] Scott AV, Stonemetz JL, Wasey JO, Johnson DJ, Rivers RJ, Koch CG, Frank SM (2015). Compliance with the Surgical Care Improvement Project for body temperature management (SCIP Inf-10) is associated with improved clinical outcomes. Anesthesiology.

[36] Campbell G, Alderson P, Smith AF, Warttig S (2015). Warming of intravenous and irrigation fluids for preventing inadvertent perioperative hypothermia. Cochrane Database Syst Rev.

[37] John M, Crook D, Dasari K, Eljelani F, El-Haboby A, Harper CM (2016). Comparison of resistive heating and forced-air warming to prevent inadvertent perioperative hypothermia. Br J Anaesth.

[38] De Witte J, Sessler DI (2002). Perioperative shivering: physiology and pharmacology. Anesthesiology.

[39] Witt L, Dennhardt N, Eich C, Mader T, Fischer T, Bräuer A, Sümpelmann R (2013). Prevention of intraoperative hypothermia in neonates and infants: results of a prospective multicenter observational study with a new forced-air warming system with increased warm air flow. Paediatr Anaesth.

